# Shifting trends in microbial keratitis following penetrating keratoplasty in Taiwan

**DOI:** 10.1097/MD.0000000000005864

**Published:** 2017-02-03

**Authors:** Hung-Chi Chen, Chia-Yi Lee, Hung-Yu Lin, David Hui-Kang Ma, Phil Yeong-Fong Chen, Ching-Hsi Hsiao, Hsin-Chiung Lin, Lung-Kun Yeh, Hsin-Yuan Tan

**Affiliations:** a Department of Ophthalmology, Chang Gung Memorial Hospital, Linkou; b Department of Medicine, Chang Gung University College of Medicine, Taoyuan; c Center for Tissue Engineering, Chang Gung Memorial Hospital, Linkou; d Department of Ophthalmology, Show Chwan Memorial Hospital, Changhua; e Institute of Medicine, Chung Shan Medical University, Taichung, Taiwan; f Department of Optometry, Chung Shan Medical University, Taichung; g Department of Optometry, Yuanpei University of Medical Technology, Hsinchu, Taiwan.; h Department of Chinese Medicine, Chang Gung University College of Medicine, Taoyuan, Taiwan.

**Keywords:** microbial keratitis, penetrating keratoplasty, shifting trends, Taiwan

## Abstract

To investigate the clinical and microbiological profiles from microbial keratitis following penetrating keratoplasty (PKP) in a tertiary referral center in Taiwan, the medical records of 648 consecutive patients (648 eyes) undergoing PKP between January 2003 and December 2007 were retrospectively reviewed. Patients who subsequently sustained microbial keratitis were enrolled and analyzed for potential risk factors, clinical manifestations, microbiological profiles, complications, graft survival, and final visual outcome. A total number of 42 corneal graft infections (6.5%) were recruited. Mean interval between corneal transplantation and graft infection was 12 ± 9.5 months. Potential risk factors included suture-related problems (31.0%), lid abnormalities (23.8%), persistent epithelial defect (23.8%), contact lens use (14.3%), dry eye (11.9%), and prior rejection episodes (4.8%). Lesions were discovered mostly at the donor-recipient junction ([DRJ] 45.2%). Positive cultures were identified in all of the morbid eyes, of which *Pseudomonas aeruginosa* was the most common pathogen (38.1%). Despite mandatory hospitalization and topical fortified antibiotics management, complications ensued such as graft failure (71.4%), hypopyon (21.4%), corneal perforation (14.3%), wound dehiscence (11.9%), and endophthalmitis (4.8%). The visual outcome was dismal that graft clarity was achieved in only 12 eyes (28.6%), and that final visual acuity deteriorated to less than 20/200 in 28 eyes (66.7%). In conclusion, microbial keratitis following PKP is a devastating event that severely impairs graft survival rate and postoperative visual outcome which usually occur within the first postoperative year. The incidence of post-PKP microbial keratitis has generally decreased in recent years whilst *P. aeroginosa* prevails as the leading cause of graft infection in our hospital. Close follow-up by ophthalmologists and elevated self-awareness of patients for at least one year are always encouraged to prevent late-onset infection.

## 1 Introduction

Microbial keratitis following penetrating keratoplasty (PKP) is a potentially devastating event necessitating urgent therapeutic intervention to prevent graft failure and subsequent poor visual outcome. Incidence of the infection ranges from 1.5% to 12.1% worldwide.^[1–8]^ Infection due to contaminated donor tissue or intraoperative contamination is rare and is usually noted soon after PKP.^[9]^ The environment accounts for the most common source of microbial keratitis after PKP.^[1,5,7,9]^ Transplanted corneas are more susceptible than normal ones to infections for several reasons, such as prolonged retention of sutures and inevitable long-term use of corticosteroids.^[10]^ Many other factors have also been associated with the development of microbial keratitis following PKP.^[11]^

Up to now only 2 studies,^[5,7]^ both conducted before year 2000, concerning microbial keratitis following keratoplasty were reported from Taiwan. The purpose of this study was to present an updated survey on graft infection after corneal transplantation and to illustrate the changing trends regarding clinical and microbiological profiles over time at our institution in Taiwan.

## 2 Materials and methods

### 2.1 Patients and ethnic declarations

This study was approved by Institutional Review Board at the Chang Gung Memorial Hospital, Linkou, Taiwan (Registration No. 96-0035B). The medical records of 648 consecutive patients (648 eyes) undergoing PKP during a 5-year period (January 2003 to December 2007) at the Chang Gung Memorial Hospital were reviewed retrospectively. Only those with a discharge diagnosis of microbial keratitis in the graft and whose microbiologic culture results were positive for bacteria, fungi, or acanthamoeba were enrolled. The exclusion criteria were sterile ulcer, clinically suspected viral keratitis, and follow-up less than 1 month. All the managements and interventions performed in this study involving human patient are adhered to the Declaration of Helsinki in 1964 and its later amendments.

### 2.2 Microbiological investigation

All patients manifesting with corneal infiltrates after PKP were subjected to corneal scrapings obtained from the peripheral and central areas of the infiltrate. Cultures were performed on chocolate agar, sheep blood agar, and thioglycolate broth and were incubated at 37 °C. Sabouraud agar plates were obtained and maintained at 25 °C to enhance fungal growth. Selective media of aerobic and anaerobic bacteria, atypical mycobacteria, and acanthamoeba were also used in patients with suggestive clinical characteristics. Gram-stained smears were performed as a routine. Positive microbial cultures were defined as growth of the same pathogen on 2 or more culture media or as growth on 1 medium of pathogens seen on stained smears of corneal scrapings. Positive fungal culture was defined on morphology.^[12]^

### 2.3 The protocol of treatment

The protocol for initial therapy consisted of immediate admission and administration of hourly broad-spectrum topical antibiotic coverage pending the results of the preliminary microbiological investigation, despite the subtle variations of choices amongst the staffs of the Corneal Service. Topical hourly antibiotics consisting of vancomycin (25 mg/mL) or cefazolin (25 mg/mL) and amikacin (25 mg/mL) or ceftazidime (25 mg/mL) and antifungal eye drops consisting of natamycin 5% (Alcon, Fort Worth, TX) or amphotericin B (0.5 mg/mL) were used as an initial empirical treatment. Subsequent modification of the medication was made according to the results of bacterial cultures and sensitivities. The frequencies of the topical antibiotics, as well as the addition of topical corticosteroids, were adjusted according to the clinical progression (resolution of the infiltrate and epithelialization of the cornea) at the discretion of each clinician.

### 2.4 Demographic and clinical data

A standardized protocol for record of each eye was directed to the following: age, sex, laterality, indications for PKP, interval between PKP and onset of keratitis, potential risk factors for microbial keratitis, lesion characteristics, microbiologic profiles, complications, graft survival, and visual outcome.

Among the risk factors, a suture-related infection was specified should an abscess was present at a suture site. Persistent epithelial defect was defined as presence of epithelial defect for more than 2 weeks. Dry eyes were characterized by a Schirmer I test of less than 10 mm.

Lesion characteristics were outlined by the size and the location. The lesion size was measured as the longest diameter, while the location was marked out as either at the center, periphery, or donor–recipient junction (DRJ).

Microbiological profiles covered both culture results and resistance to antibiotics. A positive culture was defined as growth of more than 1 colony of an organism in the inoculating streak of any culture medium.

A clear graft was defined as one with intact epithelium and free of stromal edema, allowing a clear view of iris details. A graft was hence considered failed if iris details were not clear.

## 3 Results

### 3.1 Patient demography

During the study period from January 2003 to December 2007, 42 episodes of microbial keratitis were found among 648 PKP patients, resulting in an incidence of 6.5%. The average age at presentation was 49.1 ± 21.5 years (range: 17–81 years). There were 17 females (40.5%) and 25 males (59.5%). The laterality was equally distributed with 23 left eyes (54.8%) and 19 right eyes (45.2%) involved.

The indications for the original PKP are listed in Table 1. The most commonly registered preoperative diagnosis, in order of decreasing frequency, were regraft (40.5%), aphakic bullous keratoplasty or pseudophakic bullous keratoplasty (23.8%), and traumatic or postinfectious corneal scarring (23.8%).

**Table 1:**
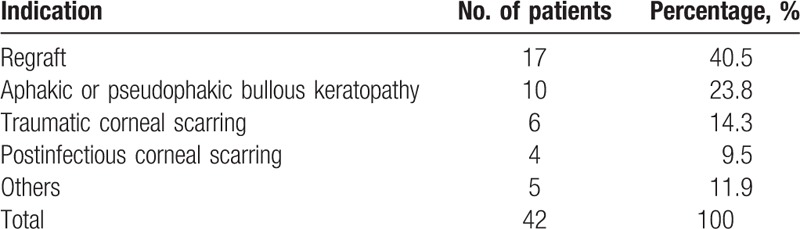
Indications for penetrating keratoplasty in 42 patients with microbial keratitis.

### 3.2 Interval between PKP and microbial keratitis

The mean interval between PKP and microbial keratitis was 12.0 ± 9.5 months (range: 1–45 months). Thirty episodes of infection (71.5%) happened beyond the 1st 6 months after PKP, with only 12 events (28.5%) within the 1st 6 months (Table 2).

**Table 2:**
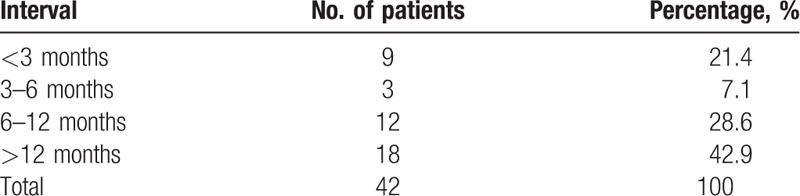
Interval between penetrating keratoplasty and microbial keratitis.

### 3.3 Potential risk factors

Potential risk factors have been recognized into 6 major groups and are shown in Table 3. All of the 13 events of suture-related infections (31.0%) emerged in the graft periphery, and all of the contact lenses used in 6 eyes (14.3%) were for therapeutic purposes. Lid abnormalities contained blepharitis, trichiasis, and lagophthalmos.

**Table 3:**
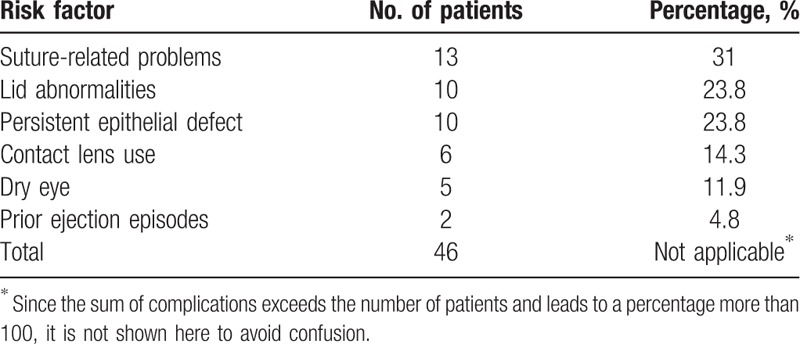
Potential risk factors for microbial keratitis following penetrating keratoplasty.

### 3.4 Lesion characteristics

The characteristics of the lesions are categorized according to the location and size, which are summarized in Table 4. The centrally located infiltrates were predominantly (62.5%) the larger ones (≥4 mm), while the infiltrates located in the periphery (86.7%) of the grafts or at the DRJ (68.4%) were largely the smaller ones (<4 mm). In terms of location, DRJ was the most common sites (19 eyes), compared to center (8 eyes) and periphery (15 eyes).

**Table 4:**
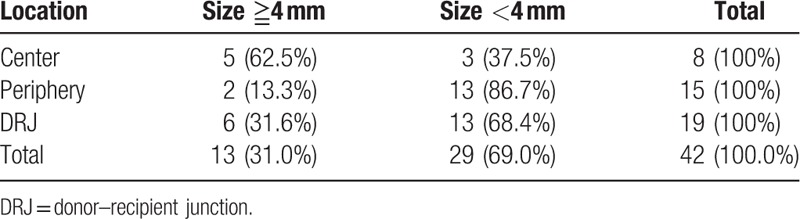
Lesion characteristics in microbial keratitis following penetrating keratoplasty.

### 3.5 Microbiologic findings and resistance to antibiotics

Pathogens identified are documented in Table 5. In the morbid 42 eyes, cultures were all positive and only 1 pathogenic organism was identified in each specimen submitted for microbiologic investigation. A broad spectrum of pathogens resulted in the development of graft infection, yet the most common pathogen identified was *Pseudomonas aeruginosa* (38.1%). Twelve of 42 (28.6%) isolates demonstrated resistance to antibiotics. Five resistant strains occurred in 10 gram-positive infections (50.0%), 5 other strains in 21 gram-negative infections (23.8%), and the rest 2 in 11 fungal infections (18.2%).

**Table 5:**
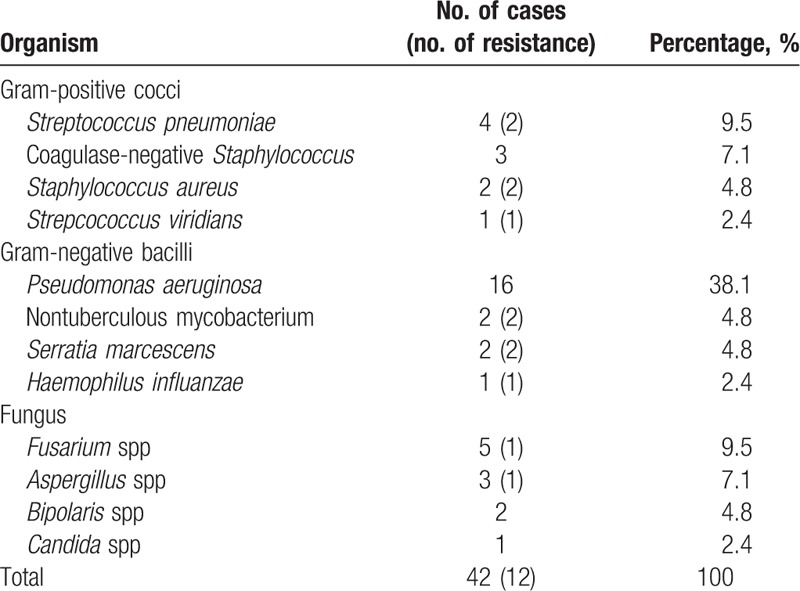
Pathogenic organisms in microbial keratitis following penetrating keratoplasty.

### 3.6 Complications

Major complications associated with graft infection are displayed in Table 6. Graft failure outnumbered all other complications and alone accounted for 71.4% (30 eyes). Hypopyon was encountered in 9 patients (21.4%).

**Table 6:**
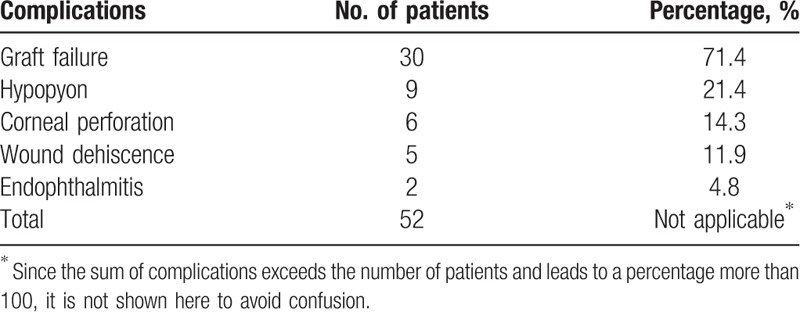
Complications in microbial keratitis following penetrating keratoplasty.

There were 6 patients experiencing corneal perforation, and 4 of them received emergent therapeutic PKP utilizing glycerin-preserved corneas.^[13]^ The rest 2 perforated eyes were applied with cyanoacrylate tissue adhesive and therapeutic soft contact lens. In all of the 6 patients, standard PKP was carried out afterwards.

At the DRJ, wound dehiscence resulting from the infection appeared in 5 patients. Unfortunately, 2 eyes developed endophthalmitis. Although repeated intravitreal injections of potent antibiotics were given, both eyes ended up with phthisis bulbi.

### 3.7 Visual outcome

Since legal blindness is generally defined as visual acuity of 20/200, we took 20/200 as a cutoff value to evaluate the visual outcome. At last follow-up, visual acuity regained over 20/200 was seen in 14 eyes, which comprised the total 12 clear grafts and 2 failed ones. As for the other 28 eyes (66.7%), visual acuity deteriorated to less than 20/200 was detected.

## 4 Discussion

For comparisons between the current and previous studies, Table 7 with important demographic and clinical parameters is provided for the following discussions.

**Table 7:**
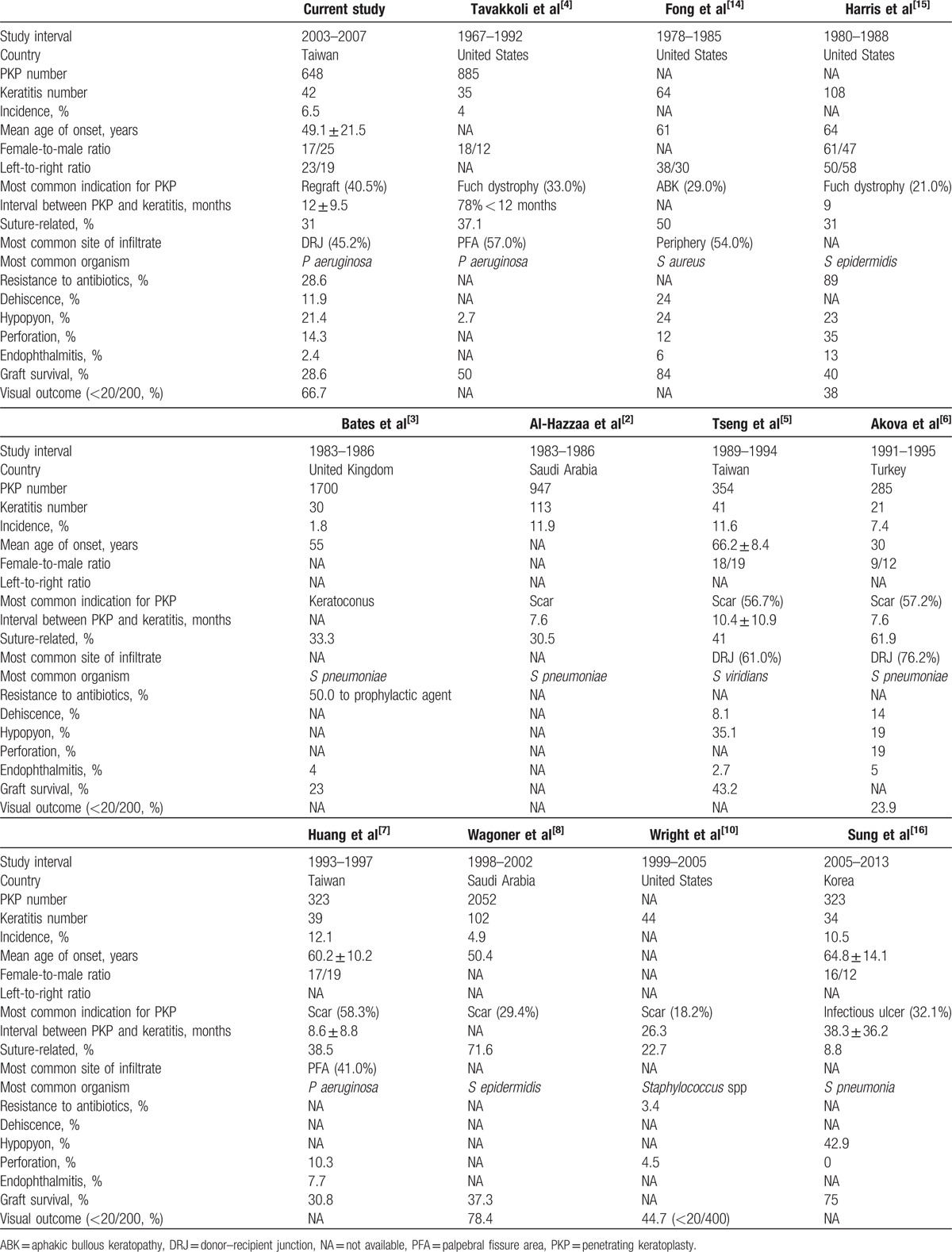
Microbial keratitis following penetrating keratoplasty: current study and published reports.

In this study, we have reported microbial keratitis following PKP in one of Taiwan largest tertiary referral centers during a 5-year period from January 2003 to December 2007. The incidence was 6.5% and the most common indication was regraft. Compared with previous works, it showed a decreasing tendency in both incidence and the percentage of corneal scarring as the indication for PKP.^[2,4–6,8,10,16]^ In a previous study recruiting patients from the same service between January 1993 and December 1997, we found that the incidence was 12.1% and that the leading indications for PKP were postinfectious corneal scarring (30.6%), traumatic corneal scarring (27.8%), and aphakic or pseudophakic bullous keratopathy (19.4%). In the current study, the incidence declined and the leading indications shifted to regraft (40.5%), aphakic bullous keratoplasty or pseudophakic bullous keratopathy (23.8%), and traumatic corneal scarring (14.3%).^[7]^

We reckon that the mean age of patients and the indications of PKP should be 2 determining factors for the decreasing incidence of corneal graft infection. Higher incidences (eg, greater than 10%) were all recorded in patient groups with mean age older than 60 years old.^[5,7,16]^ Generally, the older patients have more risk factors of infectious keratitis such as blepharitis, ocular surgery, and systemic illness.^[17]^ In addition, graft infections are apt to occur or recur given the high percentage of postinfectious corneal scarring as a leading indication in other institutions,^[2,5,6,8,10]^ and also in our previous report.^[7]^ The indications of PKP denoted in such series as featured by microbial keratitis may not reflect the true indications in general surveys for PKP. Interestingly, according to our unpublished data regarding consecutive cases for PKP, regraft was also revealed to be the leading indication for PKP (HCC, MD, PhD, unpublished data, December 2016).

Discrepancy exists among the time interval between PKP and microbial keratitis. Most studies have cited that graft infections usually occur within the 1st postoperative year, and advocated intensive follow-up of the patients for the 1st year postoperatively.^[1,2,4–6,11,15]^ However, our study disclosed an interval longer than 1 year, only shorter than 26.3 and 38.3 months reported by Wright and Afshari^[10]^ and Sung et al,^[16]^ while the reason for the remarkably long interval remained unclear. Regarding the study conducted earlier from our institution, the mean interval was 8.6 months,^[7]^ a much shorter period than that in the current study. Compared to the study by Huang et al,^[7]^ young age may be the main reason of late infections in our cases, but not in the study reported by Akova et al.^[6]^ We speculate that the younger patients, compared to the aged, are more sensitive to ocular alterations physically and more resistant to pathogens immunologically. Since more than a half of cases in both long-interval series and nearly half in ours (42.9%) experienced infection more than one year after transplantation,^[10,16]^ constant attention postoperatively is always advocated.

Several potential risks factor for post-PKP microbial keratitis have been proposed such as suture-related problems, persistent epithelial defect, and ocular surface disorder.^[11]^ The most prominent risks factor in our study was suture-related problem (31.0%), which is also a major risks factor in other studies which generally account for above 30% and can up to 71.6% in the study conducted by Wagoner et al.^[2–6,8,15]^ However, 1 study from Korea demonstrated a much lower incidence of 8.8%,^[16]^ which due to aggressive suture management and frequent follow-ups in the 1st postoperative year by their announcement. Compared our current study with previous one by Huang et al,^[7]^ the incidence of suture-related problem in their study was 38.5% which higher than our study. Maybe the much worse lid hygiene condition, which account for 43.6% of risks factor in their study but absent in our study, lead to more susceptibility to suture problem.^[7]^ Other incidence of risks factors is compactible between the current study and our previous one by Huang et al^[7]^ and our study, except decreasing in dry eye incidence (from 28.2% to 11.9%).

The most common occurred site of microbial keratitis following PKP was reported to be DRJ by Akova et al (76.2%) and Tseng and Ling (61.0%).^[5,6]^ In the current study, infection at the DRJ was found in 19 patients (45.2%), also a dominant number compared to lesions at the central and peripheral corneas (19.0% and 35.7%). Weaken structures and artificially created wounds may account for the vulnerability of DRJ to microbial infection. However, Tavakkoli and Sugar^[4]^ demonstrated that palpebral fissure area (PFA) is the most common site for microbial keratitis (57.0%). One possible explanation was that PFA is directly exposed to the external environment and may also involve part of the DRJ. A previous report from our hospital also found that the most common site of microbial keratitis is PFA (41.0%),^[7]^ the exact rate of DRJ infection was not mentioned. As for the characteristics of lesion size, lesions smaller than 4 mm in diameter were more common in our study (69.0%), while those larger than 4 mm tend to develop in the central area (38.5%). Recently, by measuring the 2 dimensions with graduated slit beam, Sung et al^[16]^ discovered that the mean size of infiltration is 15.3 mm^2^ which is also compatible with our results, given the relation between diameter and area.

Many studies have reported gram-positive cocci as the most common organisms responsible for graft infection.^[2,3,5,6,8,10,14,15]^*P aeroginosa* was the most often isolated among gram-negative ulcers,^[4,18]^ and has continued to be the prevailing etiology both in the previous and current series at our institution.^[7]^ Even in a general survey of infectious keratitis (2003–2012), we also reported the similar trend.^[19]^ The occurrence of *P aeroginosa* as a cause of corneal ulcer is frequently associated not only with extended contact lens wear,^[20,21]^ but also with more tropical climates, and with patients that are either debilitated or hospitalized.^[22]^ It began to decline as a causative pathogen during the 1990s presumably related to widespread use of disposable soft contact lenses and availability of topical fluoroquinolones.^[23,24]^ We hypothesize that extended wear of therapeutic soft contact lenses, warmer climate, and restricted 1st-line use of topical fluoroquinolones in Taiwan should be the major causes for the continued popularity of *P aeroginosa* among the microbial keratitis following PKP spanning more than a decade.

Under some circumstances, organisms that are not normally considered pathogenic may become opportunistic in a compromised eye. Overall, fungal infections have been documented variably in 1.3% to 36.1% of cases of microbial keratitis after PKP,^[3,5,8,14,15,25]^ and our study revealed an incidence within the range (18.2%). In the early 1980s’ series reported by Tuberville and Wood,^[1]^ none of the patients receiving empirical antibiotics were culture-positive for a resistant organism, and 95.0% of the bacteria tested were sensitive to gentamicin. Surprizingly, in later series, an alarming 50.0% to 89.0% of pathogens exhibited resistance to the prophylactic antibiotics used.^[3,14,15]^ This difference may reflect the emergence of resistant strains in recent years.

Several complications were recorded in our study and, of which, graft failure outweighed all the rest complications (71.4%). The rest complications included hypopyon (21.4%), corneal perforation (14.3%), wound dehiscence (11.9%), and endophthalmitis which lead to phthisis bulbi (4.8%). If compared with other studies, the graft survival rate was relatively low both in our study and that reported by Bates et al,^[3]^ but not in others.^[4,5,8,14–16]^ On the other hand, the incidence of complications such as hypopyon, corneal perforation, wound dehiscence, and endophthalmitis was similar between ours and other studies.^[3–5,7,10,14–16]^ Since regraft was the most common preoperative diagnosis in our study which leads to a surged risk of graft failure,^[26]^ it is predictable that our study has a higher ratio of graft failure. The incidence of corneal perforation in our previous study by Huang et al^[7]^ was lower than that of the current study (10.3%), but poorer prognosis was reported in patients sustaining corneal perforation, in which 3 patients later underwent repeated PKP and 1 turned out to be phthisical. In addition, the incidence of endophthalmitis was also higher in the previous study (7.7%) with all 3 patients ending up with evisceration.^[7]^ Perhaps older age, poorer lid hygiene and higher incidence of dry eye in the previous study lead to a worse prognosis.

The visual outcome is usually guarded once microbial keratitis develops after PKP with a lower rate of graft survival.^[11]^ In this study, only 28.6% of patients preserved clear grafts after keratitis and the visual acuity of 66.7% patients were seriously impaired (<20/200) with 2 eyes ended up with phthisis bulbi despite optimal antimicrobial therapy. The reported percentage of preserved graft clarity grossly ranges from 23.0% to 51.0%,^[3–5,8,15,27]^ and more than one-fifth of cases would have a final visual outcome of less than 20/200 which defined as legal blindness.^[6,8,10,15]^ Explanations for the poor prognosis after receiving appropriate antimicrobial treatment in our study are because of postinfection scarring and opacity, and the severe inflammation as well as wound dehiscence would lead to decompensation of graft endothelium.^[28]^ In our previous report,^[7]^ the graft survival rate (30.8%) was similar to the current result. However, visual acuity better than 20/200 was resumed in the total 12 clear grafts in the current series while visual acuity worse than 20/200 was noted in 3 of the 12 patients with clear grafts in previous series.^[7]^ Except for the disadvantages concerning age as well as risks factors and the fact that the clear graft cannot always promise good visual outcome,^[29]^ the definition of clear graft was not illustrated in the article written by Huang et al which may influence the analysis.

In conclusion, the incidence of post-PKP microbial keratitis has generally decreased in recent years while the risks factors, lesion characteristics, and major complications remain unchanged. Compared to the previous report from our institution, *P aeroginosa* persisted as the leading cause of graft infection but the overall incidences of post-PKP keratitis and severe complications have reduced. Close follow-up by ophthalmologists for at least 1 year after surgery, lid hygiene, and self-awareness of potential complications in post-PKP patients are highly recommended.

## References

[1] Tuberville AW Wood TO Corneal ulcers in corneal transplants. Curr Eye Res 1981;1:479–85. 7037312 10.3109/02713688109019989

[2] Al-Hazzaa SA Tabbara KF Bacterial keratitis after penetrating keratoplasty. Ophthalmology 1988;95:1504–8. 3062524 10.1016/s0161-6420(88)32988-x

[3] Bates AK Kirkness CM Ficker LA Microbial keratitis after penetrating keratoplasty. Eye (Lond) 1990;4:74–8. 2323481 10.1038/eye.1990.8

[4] Tavakkoli H Sugar J Microbial keratitis following penetrating keratoplasty. Ophthalmic Surg 1994;25:356–60. 8090413

[5] Tseng SH Ling KC Late microbial keratitis after corneal transplantation. Cornea 1995;14:591–4. 8575180

[6] Akova YA Onat M Koc F Microbial keratitis following penetrating keratoplasty. Ophthalmic Surg Lasers 1999;30:449–55. 10392732

[7] Huang SC Wu SC Wu WC Microbial keratitis – a late complication of penetrating keratoplasty. Trans R Soc Trop Med Hyg 2000;94:315–7. 10975009 10.1016/s0035-9203(00)90338-9

[8] Wagoner MD Al-Swailem SA Sutphin JE Bacterial keratitis after penetrating keratoplasty: incidence, microbiological profile, graft survival, and visual outcome. Ophthalmology 2007;114:1073–9. 17275089 10.1016/j.ophtha.2006.10.015

[9] Wilson SE Kaufman HE Graft failure after penetrating keratoplasty. Surv Ophthalmol 1990;34:325–56. 2183380 10.1016/0039-6257(90)90110-h

[10] Wright TM Afshari NA Microbial keratitis following corneal transplantation. Am J Ophthalmol 2006;142:1061–2. 17157593 10.1016/j.ajo.2006.06.051

[11] Vajpayee RB Boral SK Dada T Risk factors for graft infection in India: a case–control study. Br J Ophthalmol 2002;86:261–5. 11864877 10.1136/bjo.86.3.261 PMC1771032

[12] Chen HC Tan HY Hsiao CH Amniotic membrane transplantation for persistent corneal ulcers and perforations in acute fungal keratitis. Cornea 2006;25:564–72. 16783145 10.1097/01.ico.0000227885.19124.6f

[13] Yang JW Lin HC Hsiao CH Therapeutic penetrating keratoplasty in severe infective keratitis using glycerol-preserved donor corneas. Cornea 2012;31:1103–6. 22854559 10.1097/ICO.0b013e31821c9ba2

[14] Fong LP Ormerod LD Kenyon KR Microbial keratitis complicating penetrating keratoplasty. Ophthalmology 1988;95:1269–75. 3062538 10.1016/s0161-6420(88)33036-8

[15] Harris DJ Jr Stulting RD Waring GO 3rd Late bacterial and fungal keratitis after corneal transplantation. Spectrum of pathogens, graft survival, and visual prognosis. Ophthalmology 1988;95:1450–7. 3067181 10.1016/s0161-6420(88)33008-3

[16] Sung MS Choi W You IC Factors affecting treatment outcome of graft infection following penetrating keratoplasty. Korean J Ophthalmol 2015;29:301–8. 26457035 10.3341/kjo.2015.29.5.301 PMC4595255

[17] van der Meulen IJ van Rooij J Nieuwendaal CP Age-related risk factors, culture outcomes, and prognosis in patients admitted with infectious keratitis to two Dutch tertiary referral centers. Cornea 2008;27:539–44. 18520502 10.1097/ICO.0b013e318165b200

[18] Alexandrakis G Alfonso EC Miller D Shifting trends in bacterial keratitis in south Florida and emerging resistance to fluoroquinolones. Ophthalmology 2000;107:1497–502. 10919897 10.1016/s0161-6420(00)00179-2

[19] Hsiao CH Sun CC Yeh LK Shifting trends in bacterial keratitis in Taiwan: A 10-year review in a tertiary-care hospital. Cornea 2016;35:313–7. 26764878 10.1097/ICO.0000000000000734

[20] Mondino BJ Weissman BA Farb MD Corneal ulcers associated with daily-wear and extended-wear contact lenses. Am J Ophthalmol 1986;102:58–65. 3728625 10.1016/0002-9394(86)90210-2

[21] Cohen EJ Laibson PR Arentsen JJ Corneal ulcers associated with cosmetic extended wear soft contact lenses. Ophthalmology 1987;94:109–14. 3472135 10.1016/s0161-6420(87)33491-8

[22] Hazlett LD Corneal response to *Pseudomonas aeruginosa* infection. Prog Retin Eye Res 2004;23:1–30. 14766315 10.1016/j.preteyeres.2003.10.002

[23] Alfonso E Mandelbaum S Fox MJ Ulcerative keratitis associated with contact lens wear. Am J Ophthalmol 1986;101:429–33. 3963102 10.1016/0002-9394(86)90641-0

[24] Varaprasathan G Miller K Lietman T Trends in the etiology of infectious corneal ulcers at the F. I. Proctor Foundation. Cornea 2004;23:360–4. 15097130 10.1097/00003226-200405000-00009

[25] Smith SG Lindstrom RL Nelson JD Corneal ulcer-infiltrate associated with soft contact lens use following penetrating keratoplasty. Cornea 1984;3:131–4. 6399234

[26] Fasolo A Capuzzo C Fornea M Risk factors for graft failure after penetrating keratoplasty: 5-year follow-up from the corneal transplant epidemiological study. Cornea 2011;30:1328–35. 21926910 10.1097/ICO.0b013e318206895a

[27] Constantinou M Jhanji V Vajpayee RB Clinical and microbiological profile of post-penetrating keratoplasty infectious keratitis in failed and clear grafts. Am J Ophthalmol 2013;155:233–7. 23111174 10.1016/j.ajo.2012.07.026

[28] Davila JR Mian SI Infectious keratitis after keratoplasty. Curr Opin Ophthalmol 2016;27:358–66. 27054815 10.1097/ICU.0000000000000269

[29] Yamaguchi T Satake Y Dogru M Visual function and higher-order aberrations in eyes after corneal transplantation: how to improve postoperative quality of vision. Cornea 2015;34 (Suppl 11):S128–35. 26448170 10.1097/ICO.0000000000000589

